# Dermoscopic pattern of Kyrle's disease^☆^^☆☆^

**DOI:** 10.1016/j.abd.2019.07.007

**Published:** 2020-02-12

**Authors:** Ozlem Ozbagcivan, Banu Lebe, Emel Fetil

**Affiliations:** a Department of Dermatology, Dokuz Eylul Universitesi, Balcova, Izmir, Turkey; b Department of Pathology, Dokuz Eylul Universitesi, Balcova, Izmir, Turkey

**Keywords:** Dermoscopy, Metabolic, Pruritus, Skin diseases

## Abstract

The clinical diagnosis of Kyrle's disease may sometimes be challenging, due to the clinical similarity of lesions to other pruritic dermatosis. Although the dermoscopy is being increasingly used in daily practice, there is insufficient data in literature describing the dermoscopic patterns of Kyrle's disease, since only one report has been published to date. Herein we report our dermoscopic observation with additional diagnostic tips in a case who was diagnosed with Kyrle's disease histopathologically.

The clinical diagnosis of Kyrle's disease may sometimes be challenging, due to the clinical similarity of lesions to other pruritic dermatosis. With the increasing use of dermoscopy in daily practice, some dermoscopic patterns are useful for the recognition of this spectrum of skin disorders in recent publications.^1^, ^2^, ^3^, ^4^, ^5^, ^6^, ^7^ However, there is insufficient data in literature describing the dermoscopic patterns of Kyrle's disease, since only one report has been published to date.^1^ Herein we report our dermoscopic observation with additional diagnostic tips in a case who was diagnosed with Kyrle's disease histopathologically.

A 61-year-old women presented with a 1 month history of pruritic lesions over the whole body. Her past medical history included type 2 diabetes mellitus and end stage renal disease on peritoneal dialysis. Physical examination revealed multiple, erythematous, excoriated umbilicated papules with central adherent keratotic plugs (Fig. 1). Laboratory investigations showed elevated blood glucose and creatinine levels. A biopsy specimen obtained from the back revealed a cup shaped epidermal invagination containing degenerated collagen and elastic fibers with fibrin exudate (Fig. 2). The absence of hair structure and the presence of inflammatory debris within the invaginations were highly suggestive of Kyrle's disease. Dermoscopic examination revealed a 4-zonal concentric pattern including a crust in the center of the lesion, surrounded with a keratotic scale; a structureless whitish-gray area; a structureless pink area including dotted vessels; a structureless brown area with a peripheral scale (Fig. 3A).

**Figure 1:**
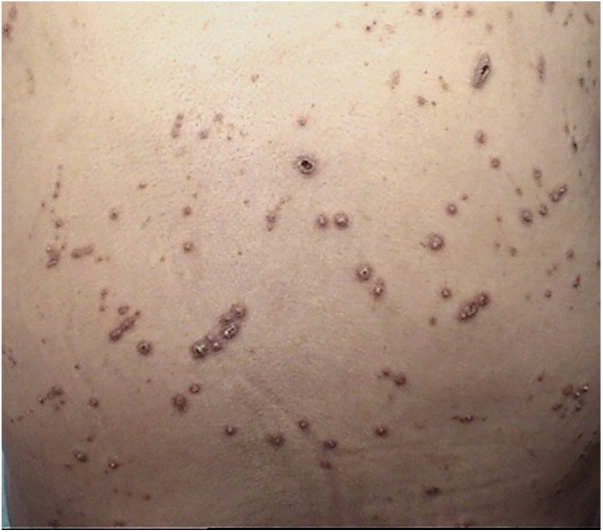
Multiple, erythematous, excoriated umbilicated papules with central adherent keratotic plugs.

**Figure 2:**
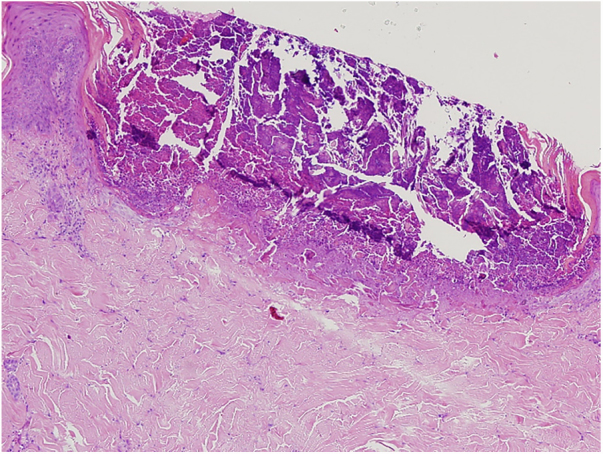
A cup shaped epidermal invagination containing degenerated collagen and elastic fibers with fibrin exudate (Hematoxylin & eosin, ×10).

**Figure 3:**
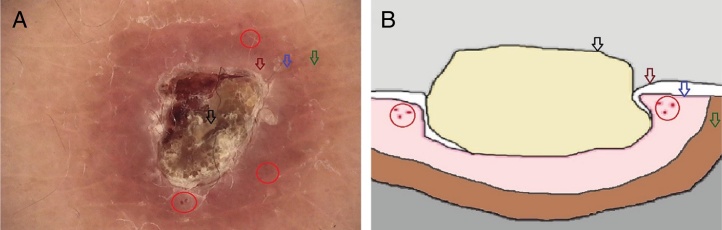
(A) Dermoscopic image of a lesion in Kyrle's disease (non-polarized dermoscopy 40×). A 4-zonal concentric pattern including a crust in the center of the lesion, surrounded with a keratotic scale (black arrow); a structureless whitish-gray area (dark-red arrow); a structureless pink area (blue arrow) including dotted vessels (red circles); a structureless brown area with a peripheral scale (green arrow). (B) Schematic illustration of the lesions in Kyrle's Disease.

Kyrle's disease is one of the rare variants of primary perforating disorders, characterized by transepidermal elimination of abnormal endogenous materials. The disease affects more commonly 30–50-year-old females, presents with pruritic hyperkeratotic and ulcerated nodules, and papules with a central keratotic plug mostly located on extensor surface of upper and lower limbs, and on the trunk. Although the exact pathogenesis remains unknown, its association has been reported sparsely with renal disorders, uremic patients on dialysis, diabetes mellitus, liver disease and paraneoplastic syndromes, tuberculosis and some fungal diseases.^8^

Over the last few years, dermoscopy has been shown to facilitate the clinical recognition of several dermatological disorders, thus reducing the number of biopsies. Recently, Russo et al. reported the dermoscopic findings of Kyrle's disease describing a 3-zonal concentric pattern, characterized by bright whitish-brownish scales in the center, a structureless whitish-gray area surrounding the central crusts, and a peripheral, brown, delicate pigmentation.^1^ In our case of Kyrle's disease, we observed a 4-zonal concentric pattern with the addition of a pink structureless area containing dotted vessels, between the structureless whitish-gray area and peripheral, brown pigmentation. Moreover, a peripheral scale surrounding the peripheral brown pigmentation was also evident. We related the central crust to the debris that fill the erosion in the epidermis; the hypopigmented structureless area to the flattening of the dermoepidermal junction by epidermal invagination; the pink structureless area with dotted vessels to the active dermal inflammation and increased vascularity during the inflammation process; and the hyperpigmented structureless area to the melanocytes, inflammatory cells and increased pigmentation of the basal keratinocytes in inflammatory processes (Fig. 3B).

In conclusion, we report an additional case of Kyrle's disease with its detailed dermoscopic features showing a strong correlation with the histological aspect of the disease. Although histopathology is still the gold standard exam for this dermatosis, the familiarity with the dermoscopic findings of perforating diseases should be increased with such reports to get more reliable clinical interpretation.

## Financial support

None declared.

## Authors’ contributions

Ozlem Ozbagcivan: Approval of the final version of the manuscript; elaboration and writing of the manuscript; intellectual participation in the propaedeutic and/or therapeutic conduct of the studied cases; critical review of the literature; critical review of the manuscript.

Banu Lebe: Approval of the final version of the manuscript; obtaining, analysis, and interpretation of the data; critical review of the manuscript.

Emel Fetil: Approval of the final version of the manuscript; critical review of the manuscript.

## Conflicts of interest

None declared.
